# A *De Novo* Transcriptome and Valid Reference Genes for Quantitative Real-Time PCR in *Colaphellus bowringi*

**DOI:** 10.1371/journal.pone.0118693

**Published:** 2015-02-18

**Authors:** Qian-Qian Tan, Li Zhu, Yi Li, Wen Liu, Wei-Hua Ma, Chao-Liang Lei, Xiao-Ping Wang

**Affiliations:** 1 College of Plant Science and Technology, Huazhong Agricultural University, Wuhan, P. R. China; 2 Hubei Insect Resources Utilization and Sustainable Pest Management Key Laboratory, Huazhong Agricultural University, Wuhan, P. R. China; University of Otago, NEW ZEALAND

## Abstract

**Background:**

The cabbage beetle *Colaphellus bowringi* Baly is a serious insect pest of crucifers and undergoes reproductive diapause in soil. An understanding of the molecular mechanisms of diapause regulation, insecticide resistance, and other physiological processes is helpful for developing new management strategies for this beetle. However, the lack of genomic information and valid reference genes limits knowledge on the molecular bases of these physiological processes in this species.

**Results:**

Using Illumina sequencing, we obtained more than 57 million sequence reads derived from *C*. *bowringi*, which were assembled into 39,390 unique sequences. A Clusters of Orthologous Groups classification was obtained for 9,048 of these sequences, covering 25 categories, and 16,951 were assigned to 255 Kyoto Encyclopedia of Genes and Genomes pathways. Eleven candidate reference gene sequences from the transcriptome were then identified through reverse transcriptase polymerase chain reaction. Among these candidate genes, *EF1α*, *ACT1*, and *RPL19* proved to be the most stable reference genes for different reverse transcriptase quantitative polymerase chain reaction experiments in *C*. *bowringi*. Conversely, *aTUB* and *GAPDH* were the least stable reference genes.

**Conclusion:**

The abundant putative *C*. *bowringi* transcript sequences reported enrich the genomic resources of this beetle. Importantly, the larger number of gene sequences and valid reference genes provide a valuable platform for future gene expression studies, especially with regard to exploring the molecular mechanisms of different physiological processes in this species.

## Introduction

Insects in a diapause status may have a stronger resistance to environmental stresses, e.g., seasonal adverse conditions and pesticides [1,2]. The cabbage beetle *Colaphellus bowringi* Baly is a serious insect pest of crucifers in mountain areas in China [3]. This beetle can survive during seasonal adverse conditions under a reproductive diapause status in soil [4,5]. Moreover, the application of pesticides is not always effective in its management [6,7]. It is well known that diapause, insecticide resistance and other physiological processes are regulated at the molecular level. However, a lack of genomic information and valid reference genes limits our understanding of the molecular bases of these physiological processes in efforts to develop new management strategies for this species.

In non-model organisms without reference genomes, abundant gene sequences can be obtained using Illumina, 454 pyrosequencing and other next-generation sequencing technologies [8–11]. Based on the identification of gene sequences, gene expression analyses performed using reverse transcriptase quantitative polymerase chain reaction (qRT-PCR) can provide new insight into complex biological process, such as in *Aedes albopictus* [12] and *Nilaparvata lugens* [13]. For example, a *de novo* analysis of the *N*. *lugens* antenna transcriptome was implemented via Illumina sequencing, and the expression of olfactory genes sequences obtained from the antenna transcriptome were analyzed by qRT-PCR [13]. Hence, prior to investigating molecular mechanisms related to diapause regulation and insecticide resistance in *C*. *bowringi*, abundant gene sequences from a *de novo* transcriptome can be obtained through Illumina sequencing.

In qRT-PCR analysis, the expression of a reference gene has been used to normalize target genes mRNA levels from the same sample [14–16]. The most common reference genes, such as *ribosomal proteins* (*RPs*), *glyceraldehyde-3- phosphate dehydrogenase* (*GAPDH*), and *actin* (*ACT*) [17–19], have been used without verification for some time [20–22]. However, the stability of these traditional reference genes varies among different insect species and experimental conditions and no reference gene can be universally used for all experimental conditions [17,23,24]. Recently, many genes, e.g., *GAPDH*, *RPs*, *ACT*, *Elongation factor-1 α* (*EF1α*), *TATA-Box binding protein* (*TBP*), *α-tubulin* (*αTUB*), and *β-tubulin* (*βTUB*), have been widely screened as valid reference genes in insect species. These genes represent different functional classes and gene families and participate in basic cellular processes [23,25,26]. In addition, four algorithms, geNorm, NormFinder, BestKeeper and RefFinder, have been commonly used to screen for valid reference genes. geNorm is an program utilized to calculate the expression stability (*M*) of candidate reference genes [14], and NormFinder is an algorithm used for the calculation of reference genes in a set of candidates [16]. BestKeeper is a Microsoft Excel-based tool that uses pair-wise correlation [27]. RefFinder can assign an appropriate weight to an individual gene and calculate the geometric mean of the weights to attain the overall final ranking [28]. Thus, according to these candidate reference genes and algorithms, valid reference genes for *C*. *bowringi* can be selected under different conditions for further gene expression by using qRT-PCR.

In the present study, Illumina sequencing technology was used to generate a substantial dataset of transcript reads of the *C*. *bowringi* transcriptome. Based on the transcriptome database, 11 candidate reference genes were verified by reverse transcriptase polymerase chain reaction (RT-PCR) and validated in different developmental stages, tissues, sexes, strains, and photoperiods to obtain the most stable reference genes for different qRT-PCR analyses in *C*. *bowringi*. The results of this study not only generate substantial sequence information but also provide valid reference genes for the analysis of gene expression. These results provide a valuable platform for future gene expression research in this species, especially for exploring the molecular mechanisms of different physiological processes, including diapause and insecticide resistance.

## Materials and Methods

### Ethics statement

The beetles used in this study were collected from a natural population from the field of Xiushui County (29°1′N, 114°4′E), Jiangxi Province, China. The field studies did not involve endangered or protected species, and no specific permissions were required for these research activities in these locations. A specimen of this beetle was deposited at the Museum of Huazhong Agricultural University.

### Experimental insects

A natural population of cabbage beetles collected in late November 2008 was maintained in-laboratory. Post-diapause adults emerging from the soil in early March 2012 (for transcriptome analysis) and October 2013 (for reference gene analysis) were reared in plastic containers (7.5 cm×7.5 cm×6 cm). The offspring of the second generation reared under conditions of 25°C and a 12:12 h light:dark photoperiod (L:D) were regarded as the high-diapause strain (HD strain) in this study. The individuals of HD strain reared at 25°C and 16:8 L:D were diapause-destined individuals while at 25°C and 12:12 L:D were non-diapause-destined individuals. A non-diapause strain (ND strain), which was not sensitive to photoperiod of diapause induction at 25°C, was established in our laboratory [29] and also used in this study. The individuals of the ND strain reared both under 16:8 L:D and 12:12 L:D at 25°C were non-diapause-destined individuals. All insects in this study were reared in illuminated incubators (SPX–250IC, Boxun Medical Instruments, Shanghai, China). The temperature was approximately 25 ± 1°C, with relative humidity at 70 ± 10%. The photoperiod was automatically controlled, with a light intensity of approximately 2.0 W/m^2^ during the photophase.

### Sample collection for transcriptome analysis

For the transcriptome analysis, 4-day-old larvae, fresh pupae, newly emerged adults without feeding, adults feeding for 2 days, and adults feeding for 5 days of four different groups were collected from the HD strain (12:12 L:D and 16:8 L:D) and ND strain (12:12 L:D and 16:8 L:D), which were reared at 25°C. The samples from each stage contained 5–10 individuals. The twenty stage samples were collected in 1.5-mL microcentrifuge tubes and immediately frozen in liquid nitrogen and stored at -80°C.

### Illumina sequencing and sequence assembly

Total RNA was isolated using an SV total RNA isolation system (Promega), according to the manufacturer’s protocol. To obtain complete gene expression information, equal amounts RNA of 20 stage samples were pooled into one sample and then used for the transcriptome analysis. Poly (A)^+^ RNA was purified using oligo [25] magnetic beads and then fragmented into short sequences at 94°C for 5 min. The cleaved poly (A)^+^ RNA was reverse-transcribed, followed by the synthesis of second-strand cDNA. After end repair and the ligation of adaptors, the products were amplified by PCR and purified using a QIAquick PCR Purification Kit (Qiagen). The cDNA library was sequenced using the Illumina sequencing platform Hiseq 2000 (Beijing Genomics Institution, Shenzhen, China). The raw reads from the images were generated using the Solexa GA pipeline 1.6.

### Unigene annotation and classification

High-quality reads were assembled into contigs using Trinity software [30]. All unigenes were used as queries to search a local protein database containing all of the protein sequences of the nr database with the BLASTX algorithm. The Clusters of Orthologous Groups (COG) classification was analyzed using annotated unigenes to search the COG database with a 10^–3^ cutoff. For a Kyoto Encyclopedia of Genes and Genomes (KEGG) pathway analysis [31], all of the unigenes were used to search the KEGG database using the default parameters. For a comparative genomics analysis, the annotated contigs were compared using BLASTP with known genes in three insect species, namely, *Drosophila melanogaster*, *Anopheles gambiae*, and *Tribolium castaneum*.

### Sample collection for reference gene analysis

Samples of different developmental stages, sexes, strains, photoperiods, and 24-h photoperiod of 4-day-old larvae were collected according to the method described in the sample collection for transcriptome analysis. According to the manufacturer’s protocol, tissue samples were collected and kept in RNAsafer (Omega Bio-tek, D13MG) to avoid RNA degradation after dissection. The experimental design and sample collection for the reference gene analysis is given in Table 1. The samples from different experimental conditions contained 5–10 individuals, and the samples for the different tissues contained 50 individuals. Each sample was prepared in three independent biological replicates. A dissection needle, Vannas scissors (54138B, 66vision Tech, Suzhou, China), and stereo-microscope (SMZ-t4, Chong Qing Optec Instrument, Chongqing, China) were used to collect the tissue samples.

**Table 1:** Experimental design and sample collection for reference genes analysis in *C*. *bowringi*.

Condition	Strain	Photoperiod (L:D)	Samples	Number of samples
Developmental stage	HD	12:12	2-, 4-, and 6-day-old larvae, 3-day-old pupae, newly emerged adults without feeding, and adults feeding for 2, 4, and 6 days	8
Tissue	HD	12:12	head, fat body, ovary, and carcass were dissected from female adults feeding for 2 days	4
Sex	HD	12:12	male and female adults feeding for 2 days	2
Strain	HD, ND	12:12	adults feeding for 2 days	2
Photoperiod	HD	16:8, 12:12	adults feeding for 2 days	2
24-h Photoperiod of 4-day-old larvae	HD	12:12	4-day-old larvae were collected at 4 h-intervals of Zeitgeber time	7

The insects used for strain analysis were collected from HD and ND strains reared at 25°C under 12:12 L:D while for photoperiod analysis were collected from HD strain reared at 25°C under 16:8 L:D and 12:12 L:D. And the insects used for additional conditions were collected from HD strain reared at 25°C under 12:12 L:D.

### Reference gene selection and primer design

Eleven reported reference genes, *GAPDH*, *RPL32e*, *RPL19*, *EF1α*, *TBP*, *TBP1*, *ACT1*, *ACT2*, *αTUB*, *αTUB1*, and *βTUBC*, were selected for evaluation in *C*. *bowringi*. These candidate reference genes represent different functional classes and gene families and have been widely used in qRT-PCR analysis in insects [18,19,23,26]. All the gene sequences were obtained by screening the transcriptome data of *C*. *bowringi*. The gene sequences were deposited in GenBank (the accession numbers are listed in S1 Table). All gene-specific primers were designed using the NCBI Primer-BLAST primer designing tool (http://www.ncbi.nlm.nih.gov/tools/primer-blast/index.cgi?LINK_LOC=BlastHome) (S1 Table).

### Total RNA extraction and cDNA synthesis for reference gene analysis

RNAsafer used in tissue samples analysis was thoroughly removed prior to RNA isolation. Total RNA was extracted using TRIzol (TaKaRa Bio., Dalian, China) following the manufacturer’s protocol. The concentration and purity of the total RNA isolated from the different samples were determined using a NanoDrop 2000 (Thermo Scientific, Wilmington, DE, USA). The 260/280 ratios ranged from 1.96 to 2.11 for all RNA samples. One μg of total RNA was used to synthesize first-strand cDNA using the PrimeScriptRT reagent kit (TaKaRa Bio, Dalian, China) with gDNA Eraser (Perfect Real Time), according to the manufacturer’s protocol. The synthesized cDNA was stored at -20°C.

### Quantitative real-time PCR analysis

Quantitative PCR reactions were performed using a MyIQ2 Two-color Real-time PCR Detection System (Bio-Rad, USA) and SYBR Premix Ex Taq II (TaKaRa, Dalian, China). The cDNA products were then diluted 20-fold with deionized water and used as templates in real-time PCR. The reaction mixture (20 μL) contained 10 μL 2 × SYBR Premix Ex Taq II, 0.4 μM each of forward and reverse primers, and 2 μL of template cDNA. The PCR amplification was performed under the following conditions: 95°C for 30 s, followed by 40 cycles of 95°C for 5 s and 60°C for 30 s. The qRT-PCR efficiency was determined for each gene using a slope analysis with a linear regression model. Relative standard curves for the transcripts were generated with serial dilutions of cDNA (1/2, 1/10, 1/50, 1/250, 1/1250). The corresponding qRT-PCR efficiencies (E) were calculated according to the equation E = (10^[-1/slope]^-1)*100 [32]. The cDNA samples were derived from each of three independent biological replicates under different experimental conditions, and all samples were analyzed by three technical replicates.

### Statistical analysis of reference gene selection

A data analysis of the quantitative real-time PCR was carried out using Bio-Rad iQ5 Optical System software (version 2.1.94.617) (Bio-Rad Laboratories, Hercules, CA, USA). The fluorescent signal of the threshold cycle (Ct value) is the initial marker of a significant difference from the background. All the biological replicates were used to calculate the average Ct value. The stability of 11 candidate reference genes was comprehensively evaluated using the algorithms geNorm version 3.5 (http://medgen.ugent.be/~jvdesomp/genorm/) [14], NormFinder version 0.953 (http://www.mdl.dk/publications normfinder.htm) [16], and BestKeeper (http://www.wzw.tum.de/gene-quantification/bestkeeper.html) [27]. Lastly, the tested candidates were compared and ranked with a web-based analysis tool, RefFinder (http://www.leonxie.com/referencegene.php) [28]. GeNorm calculated the expression stability value (*M*) of each gene and selected two most stable genes. *M* value below 1.5 indicates that the gene can be used as a reference gene and the lower *M* value means the more stable of the gene. Pairwise comparison (*Vn/Vn+1*) value was used to determine the optimal number of reference genes and pairwise comparison values below 0.15 indicate that an additional reference gene will not be required for accurate normalization. NormFinder calculated the genes Stability value (SV) and ranked the genes. The SV value is lower, the gene is more stable and one most stable reference gene can be confirmed. BestKeeper determines the genes expression stability through calculating the geometric mean (GM) and standard deviation (SD) based on all genes Ct values. SD value below 1 indicates that the gene can be used as a reference gene and the lower SD value means the more stable of the gene. Finally, RefFinder made a combination of these methods results and recommended the most suitable reference genes in different conditions.

## Results

### Illumina sequencing and *de novo* assembly

Equal amounts of RNA from 20 samples (samples of four different groups of *C*. *bowringi* described in the Materials and Methods section) were pooled for the transcriptome analysis using the Illumina sequencing platform to obtain deep coverage of the transcriptome. After quality checks, 54 million 90-bp-long reads were obtained and used for assembly with Trinity software; a total of 39,390 unigenes was obtained (Table 2). The length of 21,398 unigenes (54.32%) ranged from 100 to 500 bp, and the length of 8,658 unigenes (21.98%) ranged from 501 to 1000 bp; 5,333 unigenes (13.54%) had a length of more than 1500 bp. The mean unigene length was 792 bp (S1 Fig).

**Table 2:** Summary of the *C*. *bowringi* transcriptome.

Details	Number
Total Raw Reads	57,759,016
Total Clean Reads	54,463,364
Total clean base pairs (bp)	4,901,702,760
Total number of contigs	83,809
Total number of unigenes	39,390
Mean length of contigs (bp)	369
Mean length of unigenes (bp)	792
Unigene with E-value < 10^–5^	24,070
GC percentage	43.40%

### Annotation of predicted proteins

Of the *C*. *bowringi* transcripts, 24,070 (61.1%) showed significant similarity (E-value < 1e–5) to transcripts of known proteins in the NCBI database (Table 3). The majority of the transcripts (55%) matched to arthropod proteins, and the remainder was similar to non-insect eukaryotes proteins (5%) and bacterial proteins (0.6%). A total of 19 sequences were similar to the sequences of viral proteins, and non-insect eukaryotic proteins (Table 3).

**Table 3:** Summary of the BLASTX search of *C*. *bowringi* sequences.

Details	Number
Significant matches	24,070
Archaea	0
Arthropoda	21,662
Bacteria	252
Other eukaryotes	2,137
Viruses	19
Non-significant matches	15,320
Total	39,390

### Comparative analysis of the transcripts

**Fig 1:**
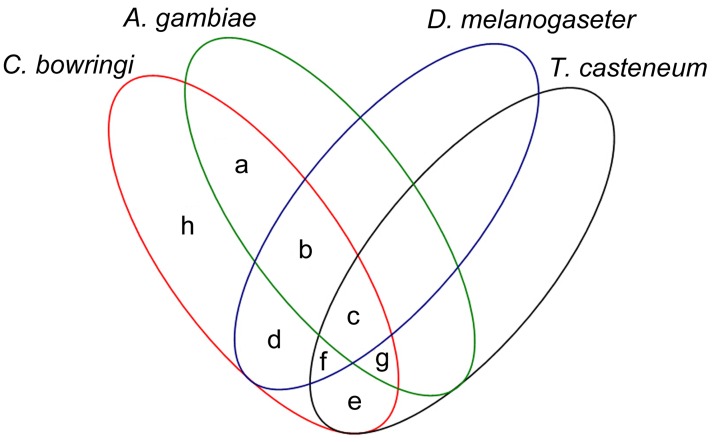
Summary of the comparisons analysis. *C*. *bowringi* transcriptomic sequences were compared with protein sequences from the draft genomes of *Anopheles gambiae*, *Drosophila melanogaster*, and *Tribolium castaneum*. a = 75; b = 222; c = 16175; d = 89; e = 4871; f = 593; g = 703; h = 16662.

The derived *C*. *bowringi* transcripts were compared with protein sequences in the existing genomes of *D*. *melanogaster*, *A*. *gambiae*, and *T*. *castaneum* using the BLASTX algorithm. A high sequence similarity (57%, 22,342 of 39,390) between *C*. *bowringi* transcripts and the *T*. *castaneum* genome was observed (Fig. 1). The similarity between *C*. *bowringi* transcripts and the *D*. *melanogaster* and *A*. *gambiae* genomes was 33% and 34%, respectively. These four insect species shared 11,419 sequences. Approximately 42% of the *C*. *bowringi* sequences (16,662 out of 39,390) did not show BLASTX similarity, which suggest that they represent non-coding RNAs, un-translated regions, non-conserved regions, or novel proteins of *C*. *bowringi* (Fig. 1).

### COG and KEGG classifications

**Fig 2:**
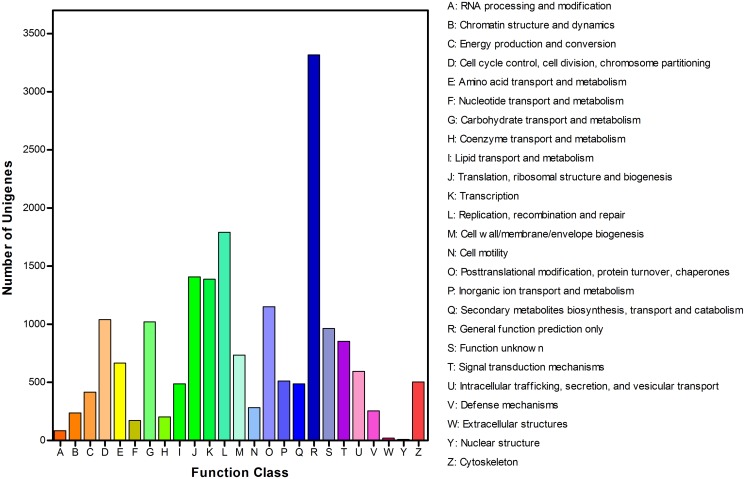
COG function classification of *C*. *bowringi* unigenes.

A total of 24,070 transcripts with matching records were obtained, and a COG classification was obtained for 9048 sequences (Fig. 2). Of the 25 COG categories, a general function prediction was identified as the largest cluster group (3317, 37%), followed by cluster groups of replication, recombination, and repair (1792, 20%) and translation, ribosomal structure, and biosynthesis (1408, 16%). Extracellular structures (20, 0.00221%) and nuclear structure (9, 0.009947%) accounted for the smallest number of sequences (Fig. 2). For a biological pathways analysis in *C*. *bowringi*, the 24,070 annotated sequences were mapped to the canonical reference pathways in KEGG. In total, 16,951 sequences were assigned to 255 KEGG pathways (S2 Table). Of these sequences, 2480 belonged to metabolic pathways, 609 to purine metabolism pathways, and 582 to pathways regulating the actin cytoskeleton.

### Identification of candidate reference genes

The sequences of the 11 candidate reference genes were screened from the *C*. *bowringi* transcriptome. The sequence accuracy of the 11 candidate reference genes was confirmed by RT-PCR, and the target RT-PCR products of the genes were sequenced again by the experts from Genscript Company (Nanjing, China). The similarity between these sequences from the transcriptome and the data obtained from Genscript was above 99.9%. The complete open reading frames (ORFs) obtained, including the partial sequences of the *RPL19* ORF, were submitted to the GenBank database (S1 Table).

### Expression profiles of candidate reference genes

For each reference gene, the primer sets amplified a unique PCR product with a single-peak dissociation curve, and all the products ranged from 80 to 137 bp. The melting temperature (Tm) of all primers for the PCR products was about 60°C. The amplification efficiency (E) of the PCR reactions ranged from 100.0% for *RPL32e* to 108.2% for *TBP1*, and the correlation coefficients (R^2^) ranged between 0.996 and 0.998, respectively (S3 Table).

**Fig 3:**
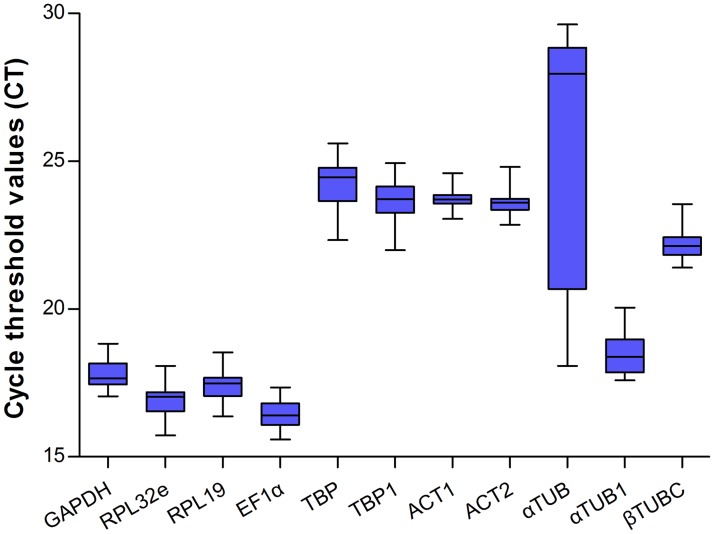
Expression levels of candidate reference genes for different samples of *C*. *bowringi*. The expression levels are displayed as cycle threshold (Ct) values of the *C*. *bowringi* reference genes used in this study. Blue bars indicate the 25/75 percentiles, whisker caps indicate the min to max, and the line denotes the median.

The expression levels of these genes were analyzed by qRT-PCR, and the Ct values showed different levels of transcript abundance. The mean Ct values of the 11 reference genes ranged from 16.44 to 25.08 cycles. Judging from the variations in Ct values, *ACT1* exhibited the lowest variation in expression (below two cycles), followed by *EF1α*, *GAPDH*, and *ACT2*, whereas *αTUB* showed the highest variation (Fig. 3).

### Analysis of gene expression stability

**Fig 4:**
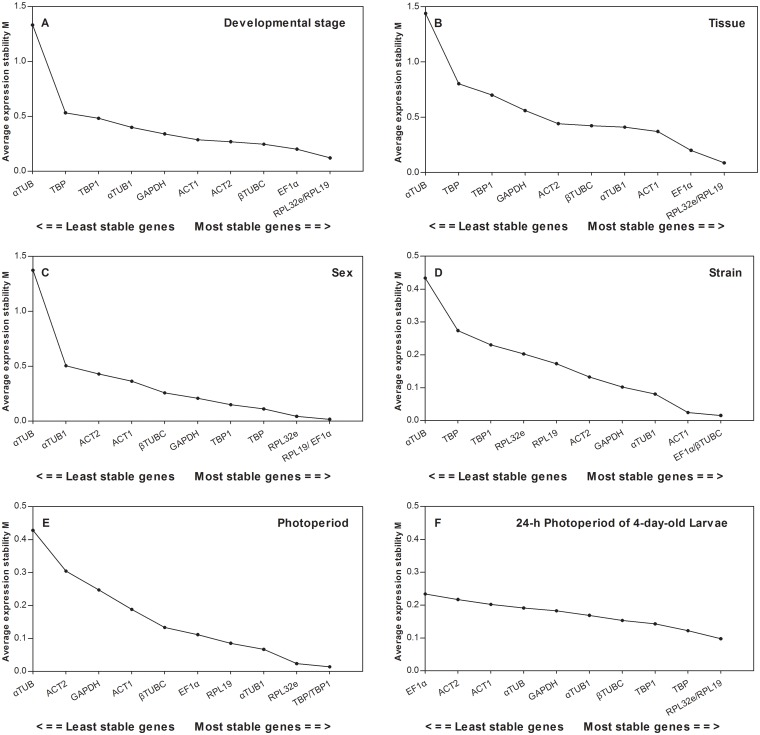
The stability of candidate reference gene expression, as calculated by geNorm. Stability of reference gene expression in the following samples of *C*. *bowringi*: A) different developmental stages; B) different tissues; C) different sexes; D) different strains; E) different photoperiods; F) 24-h photoperiod of 4-day-old larvae.

**geNorm analysis.** Based on the average *M* values, the most stable reference genes for developmental stages were *RPL32e* and *RPL19*; *αTUB* showed an *M* value higher than 1.0, indicating that its expression level was the most variable (Fig. 4A). With regard to different tissues, *RPL32e*, *RPL19*, and *EF1α* were found to be the most appropriate reference genes (Fig. 4B). *RPL19* and *EF1α* were the most stable genes for different sexes (Fig. 4C). For different strains, the *M* value of all the candidate reference genes was below 0.5, and *EF1α* and *βTUBC* were the most invariable (Fig. 4D). Regarding different photoperiods, the most stable reference genes were *TBP* and *TBP1* (Fig. 4E). Additionally, *RPL32e* and *RPL19* were the most stable genes with the lowest *M* values for the 24-h photoperiod of 4-day-old larvae, and the *M* value of *EF1α* was the highest (Fig. 4F).

**Fig 5:**
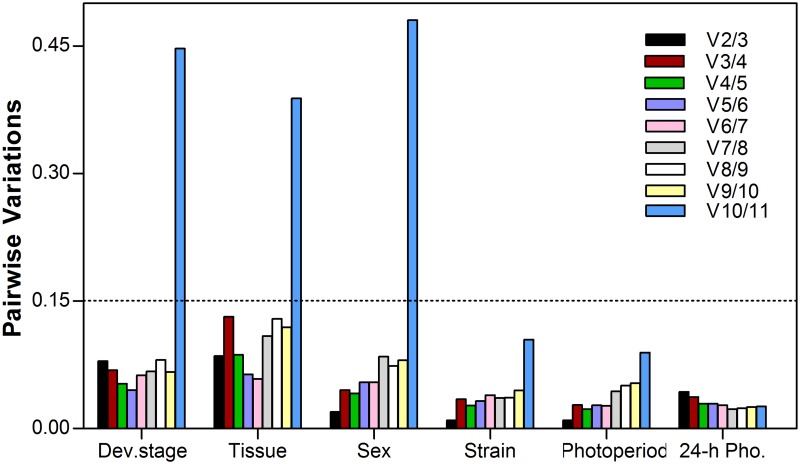
Determination of the optimal number of reference genes, as calculated by geNorm. Average pairwise variations (V) were calculated by geNorm between the normalization factors NF_n_ and NF _n+1_ to indicate whether the inclusion of an extra reference gene adds to the stability of the normalization factor. Values < 0.15 indicate that additional genes are not required for the normalization of gene expression.

To determine the optimal number of reference genes required for accurate normalization, the pairwise variation (*Vn/Vn+1*) was calculated using geNorm. In terms of developmental stage, tissue, and sex, all the *Vn/Vn+1* values, except for the *V10/11* value, were below 0.15. In addition, regarding strain and photoperiod treatment, all the *Vn/Vn+1* values were below 0.15 (Fig. 5).

**NormFinder analysis.** The results indicated that the ranking of the NormFinder reference gene stability was marginally different from that of geNorm (Table 4). For developmental stage, the most stable genes were *EF1α* and *βTUBC*, whereas the most unstable gene was *αTUB*. With regard to stability, the selected reference genes in different tissues ranked as follows: *ACT1*, *ACT2*, *αTUB1*, *EF1α*, *RPL32e*, *TBP1*, *βTUBC*, *RPL19*, *TBP*, *GAPDH*, and *αTUB*. For sex, the most stable genes were *RPL19* and *EF1α*, sharing the same stability values. *ACT1*, *βTUBC*, and *ACT2* were the most stable genes for different strains, with all the values of reference genes below 0.5. The most stable genes for photoperiod were *RPL19* and *αTUB1*. For the 24-h photoperiod of 4-day-old larvae, the stability values of the 11 reference genes were below 0.2, with little variation. With the exception of the treatment of 24-h photoperiod of 4-day-old larvae, the least stable gene was the same, namely, *αTUB*.

**Table 4:** Ranking of candidate reference genes for *C*. *bowringi* under different experimental conditions, as evaluated by NormFinder.

Rank	Development Stage		Tissue		Sex		Strain		Photoperiod		24-h Photoperiod of 4-day-old Larvae	
	**Gene**	**SV**	**Gene**	**SV**	**Gene**	**SV**	**Gene**	**SV**	**Gene**	**SV**	**Gene**	**SV**
1	*EF1α*	0.066	*ACT1*	0.060	*RPL19*	0.006	*ACT1*	0.001	*RPL19*	0.011	*RPL19*	0.034
2	*βTUBC*	0.066	*ACT2*	0.221	*EF1α*	0.006	*βTUBC*	0.001	*αTUB1*	0.011	*TBP*	0.056
3	*TBP*	0.125	*αTUB1*	0.288	*RPL32e*	0.017	*ACT2*	0.001	*EF1α*	0.017	*RPL32e*	0.087
4	*TBP1*	0.125	*EF1α*	0.331	*TBP*	0.025	*EF1α*	0.009	*βTUBC*	0.017	*αTUB1*	0.103
5	*ACT1*	0.256	*RPL32e*	0.344	*TBP1*	0.025	*GAPDH*	0.040	*RPL32e*	0.064	*βTUBC*	0.104
6	*ACT2*	0.282	*TBP1*	0.373	*GAPDH*	0.038	*TBP*	0.046	*TBP1*	0.090	*ACT1*	0.106
7	*RPL19*	0.289	*βTUBC*	0.381	*βTUBC*	0.215	*TBP1*	0.136	*TBP*	0.105	*TBP1*	0.119
8	*RPL32e*	0.376	*RPL19*	0.422	*ACT1*	0.592	*RPL19*	0.200	*ACT1*	0.206	*αTUB*	0.135
9	*GAPDH*	0.509	*TBP*	0.500	*ACT2*	0.657	*αTUB1*	0.217	*GAPDH*	0.326	*ACT2*	0.154
10	*αTUB1*	0.557	*GAPDH*	0.988	*αTUB1*	0.840	*RPL32e*	0.275	*ACT2*	0.427	*GAPDH*	0.162
11	*αTUB*	3.408	*αTUB*	2.957	*αTUB*	3.662	*αTUB*	0.448	*αTUB*	0.680	*EF1α*	0.194

The ranking of candidate reference genes for combinations of the samples was then analyzed. For different photoperiods combined with tissues, the best combination of two genes was *TBP1* and *αTUB1*, the most stable gene was *ACT1*, and the most unstable gene was *αTUB*. According to the NormFinder analysis of the combination of photoperiod with strain, the best combination of two genes was *EF1α* and *βTUBC*, the most stable genes were *βTUBC*, and the most unstable gene was *αTUB*. In the two treatments of photoperiod and 24-h photoperiod of 4-day-old larvae, the best combination of two genes was *RPL19* and *αTUB1*, the most stable gene was *RPL19*, and the most unstable gene was *αTUB* (S4 Table). The results of the NormFinder analysis indicated that the most stable genes for single factors and double factors may be different due to the expression levels of different genes.

**BestKeeper analysis.** The results of the BestKeeper analysis (S5 Table) were more or less consistent with the results obtained for geNorm and NormFinder. In the six different treatments of development stage, tissue, sex, strain, photoperiod, and 24-h photoperiod of 4-day-old larvae, the most stable genes were *ACT1*,*GAPDH*, *ACT2*, *EF1α*, *GAPDH*, and *TBP*, respectively. According to the results of the BestKeeper analysis, *αTUB* was the least stable gene.

**Fig 6:**
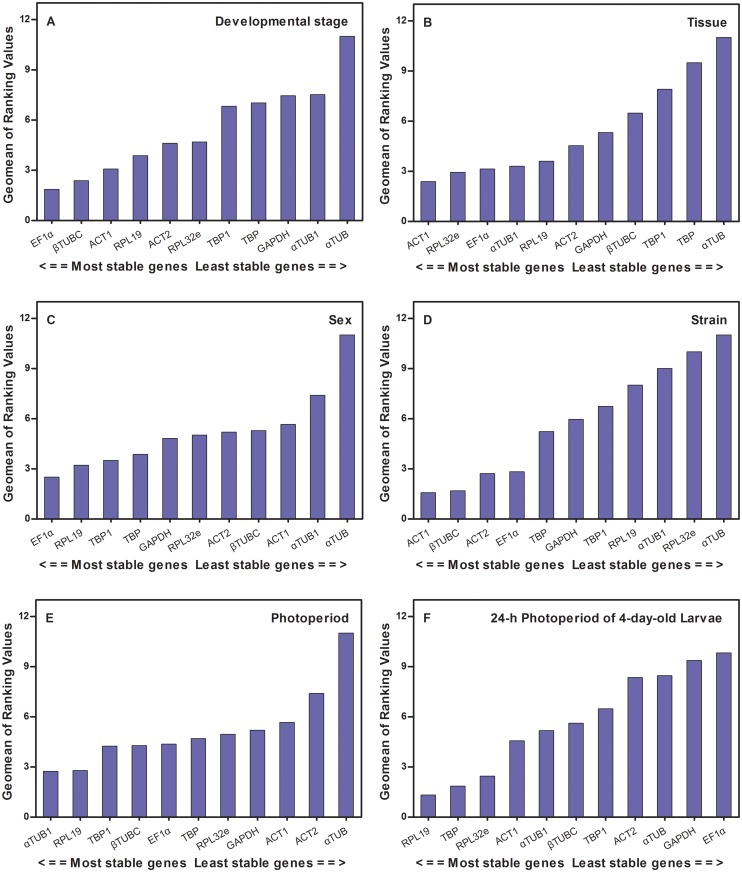
The stability of candidate reference gene expression, as calculated by the Geomean method in RefFinder. A lower Geomean ranking indicates more stable expression. Stability of reference genes in the following samples: A) different developmental stages; B) different tissues; C) different sexes; D) different strains; E) different photoperiods; F) 24-h photoperiod of 4-day-old larvae.

**RefFinder analysis.** According to the results of the RefFinder analysis, in the six different treatments of developmental stage (Fig. 6A), tissue (Fig. 6B), sex (Fig. 6C), strain (Fig. 6D), photoperiod (Fig. 6E), and 24-h photoperiod of 4-day-old larvae (Fig. 6F), the most stable genes were *EF1α*, *ACT1*, *EF1α*, *ACT1*, *αTUB1*, and *RPL19*, respectively.

## Discussion

In this study, we established a valuable platform for gene expression analysis in *C*. *bowringi*. A substantial dataset of transcript reads of the *C*. *bowringi* transcriptome was obtained using Illumina sequencing technology. The sequences of 11 candidate reference genes from this transcriptome database were confirmed by RT-PCR. This study revealed that *RPL19*, *ACT1*, and *EF1a* were the most stable reference genes, whereas *aTUB* and *GAPDH* were the least stable reference genes for different qRT-PCR analyses in *C*. *bowringi*.

### Transcriptome sequencing provides high-quality gene sequence information for *C*. *bowringi*

Abundant gene sequences can be obtained using a powerful tool: next-generation sequencing technology. In our study, Illumina sequencing was applied to *C*. *bowringi*, and 83,809 contigs with a mean size of 369 bp and 39,390 unigenes with a mean size of 792 bp were obtained. Both the quantity of numbers and sizes of contigs and unigenes obtained, indicated higher abundance of our transcriptome compared with the results of another study on diapause mechanisms in *A*. *albopictus* that used a similar sequencing technology [33]. This abundant database will provide a basis for gene expression analysis and greatly improve the study of molecular mechanisms of different physiological processes including diapause and insecticide resistance in *C*. *bowringi*.

In general, gene function analysis is necessary for investigating the molecular mechanisms of diapause regulation and insecticide resistance in *C*. *bowringi*. In our study, 24,539 unigenes were further annotated with regard to gene function: 9,048 (36.9%) sequences had a COG classification, covering 25 categories, and 16,951 (69.1%) sequences were assigned to 255 KEGG pathways. Comprehensive gene function classifications for *C*. *bowringi* were similar to the results of studies in *N*. *lugens* [34] and *Rhyacionia leptotubula* [35]. This copious biological information is helpful for understanding the fundamental molecular pathways of this beetle.

### *EF1α*, *ACT1*, and *RPL19* are the most stable reference genes for different qRT-PCR analyses in *C*. *bowringi*

qRT-PCR, with the advantages of high throughput, sensitivity, and accuracy, has been frequently used to study the molecular mechanisms of many physiological processes, such as diapause [36,37]. However, before a reference gene is used for qRT-PCR analyses, the stability of the gene should be evaluated under various conditions [20–22]. One major conclusion of our study was that appropriate reference genes (*RPL19*, *ACT1*, and *EF1a*) were found for several different experimental conditions and that *aTUB* and *GAPDH* as traditional reference genes were the least stable.

In detail, the results indicated that *RPL19*, *ACT1*, and *EF1a* showed steady expression in *C*. *bowringi* under several experimental conditions. *RPL19* had the most stable gene expression in the different sexes and photoperiod treatments. Various ribosomal proteins have also been reported to have stable expression in different developmental stages in other species, such as in *L*. *decemlineata* [26], *B*. *tabaci* [18], *B*. *mori* [24], and *Spodoptera exigua* [24]. Thus, *RPL19* could be a candidate reference gene with regard to biotic conditions in qRT-PCR analysis. In addition, *ACT1* was the most stable gene in different tissues and strains. In fact, *ACT* has been deemed as the most stable reference gene in many species, such as in *Folsomia candida* and *Orchesella cincta* [38], *Schistocerca gregaria* [39], *D*. *melanogaster* [40], and *Rhodnius prolixus* [41]. However, for *B*. *tabaci*, *ACT* was found to be unstable and was disqualified as a suitable reference gene for different tissues and hosts [18]. Thus, caution should be exercised when utilizing *ACT* because the protein plays key roles in different important cellular processes [26]. The current data showed that *EF1α* exhibited stability in different developmental stages and sexes in *C*. *bowringi*. *EF1a* has rarely been used as a normalizer but has recently been selected as a suitable reference gene in a number of species [17,19,42]. *EF1α* was the most appropriate reference gene in another coleopteran *Agrilus planipennis* [43]. Taken together, although *RPL19*, *ACT1*, and *EF1a* showed steady expression under certain experimental conditions, these genes did not present high stability under other conditions and in other insects.

In contrast, our results showed that *aTUB* and *GAPDH* were the least stable reference genes in *C*. *bowringi*. Although *aTUB1* was the most stable gene in different photoperiods and *βTUBC* was the second most stable gene in developmental stages and strains, *aTUB* was the least stable gene under all the experimental conditions (Figs. 4 and 6). Nonethelss, in *Bactrocera dorsalis*, *aTUB* was found to be much more stable than *βTUB* in all tissues and was one of the most stable genes in all samples [17]. GAPDH is an enzyme that plays an important role in energy metabolism and is often used as an endogenous control for normalization [19]. However, in the current study, *GAPDH* clearly showed reduced stability compared with other reference genes. Similarly, several previous studies have demonstrated that *GAPDH* has low expression stability under certain conditions, such as in the midgut, Malpighian tubules, and fat body of the oriental fruit fly *B*. *dorsalis* [17] and in *B*. *tabaci* under different biotic and abiotic conditions [18]. In contrast, *GAPDH* was identified as the most stable gene in the head of honeybees after bacterial challenge [44] and in different developmental stages and temperature-stressed larvae of *S*. *litura* [19]. Thus, the commonly used reference genes *aTUB* and *GAPDH* revealed unstable expression in *C*. *bowringi* and different expression stability in other insects. It suggests that the expression stability of *aTUB* and *GAPDH* should be evaluated before they are used as reference genes in the future.

## Conclusions

In this study, a comprehensive transcriptome of *C*. *bowringi* was generated using Illumina sequencing. Altogether, 83,809 contigs with a mean size of 369 bp and 39,390 unigenes with a mean size of 792 bp were obtained. After comprehensive analysis, *EF1α*, *ACT1*, and *RPL19* were found to be the most stable reference genes for different qRT-PCR analyses in *C*. *bowringi* (Table 5), whereas *aTUB* and *GAPDH* showed relatively low expression stability. This work established a platform for future gene expression studies in the cabbage beetle. The results could both benefit the investigation of gene function related to diapause regulation and insecticide resistance and also enrich organism genome resources and provide information on reference gene selection.

**Table 5:** Recommended *C*. *bowringi* reference genes for qRT-PCR under various experimental conditions.

Condition	Recommended reference genes		
Developmental stage	*EF1α*	*βTUBC*	*ACT1*
Tissue	*ACT1*	*RPL32e*	*EF1α*
Sex	*EF1α*	*RPL19*	*TBP1*
Strain	*ACT1*	*βTUBC*	*ACT2*
Photoperiod	*αTUB1*	*RPL19*	*TBP1*
24-h Photoperiod of 4-day-old larvae	*RPL19*	*TBP*	*RPL32e*

## Supporting Information

S1 Fig Length distribution of *Colaphellus bowringi* unigenes. (TIF)

S1 Table Primers used for the RT-PCR analysis to identify the accuracy of *Colaphellus bowringi* reference genes. (DOC)

S2 Table KEGG pathways of unigenes collected from *Colaphellus bowringi*. (XLS)

S3 Table Primers used for qRT-PCR analysis to explore valid reference genes of *Colaphellus bowringi*. (DOC)

S4 Table Ranking of candidate reference genes for *Colaphellus bowringi* in response to combination samples, as evaluated by NormFinder. (DOC)

S5 Table The statistical results of the candidate reference genes of *Colaphellus bowringi*, as calculated by BestKeeper. (DOC)

## References

[1] Baker MB, Porter AH. Use of sperm precedence to infer the overwintering cost of insecticide resistance in the Colorado potato beetle. Agr Forest Entomol. 2008;10: 181–187.

[2] Caron V, Myers JH. Positive association between resistance to *Bacillus thuringiensis* and overwintering survival of cabbage loopers, *Trichoplusia ni* (Lepidoptera: Noctuidae). B Entomol Res. 2008;98: 317–322. 10.1017/S0007485307005597 18257958

[3] Xue F, Li A, Zhu X, Gui A, Jiang P, Liu X. Diversity in life history of the leaf beetle, *Colaphellus bowringi* Baly. Acta Entom Sin. 2002;45: 494–498.

[4] Xue F, Spieth HR, Aiqing L, Ai H. The role of photoperiod and temperature in determination of summer and winter diapause in the cabbage beetle, *Colaphellus bowringi* (Coleoptera: Chrysomelidae). J Insect Physiol. 2002;48: 279–286. 12770101 10.1016/s0022-1910(01)00172-x

[5] Wang X, Ge F, Xue F, You L. Diapause induction and clock mechanism in the cabbage beetle, *Colaphellus bowringi* (Coleoptera: Chrysomelidae). J Insect Physiol. 2004;50: 373–381. 15121450 10.1016/j.jinsphys.2004.01.002

[6] Gao Y, Jurat-Fuentes JL, Oppert B, Fabrick JA, Liu C, Gao J, et al Increased toxicity of *Bacillus thuringiensis* Cry3Aa against *Crioceris quatuordecimpunctata*, *Phaedon brassicae* and *Colaphellus bowringi* by a *Tenebrio molitor* cadherin fragment. Pest Manag Sci. 2011;67: 1076–1081. 10.1002/ps.2149 21495115

[7] Shu C, Su H, Zhang J, He K, Huang D, Song F. Characterization of *cry9Da4*, *cry9Eb2*, and *cry9Ee1* genes from *Bacillus thuringiensis* strain T03B001. Appl Microbiol Biot. 2013;97: 9705–9713. 10.1007/s00253-013-4781-5 23455566

[8] Schuster SC. Next-generation sequencing transforms today's biology. Nat Methods. 2007;5: 16–18. 10.1038/nmeth1156 18165802

[9] Ansorge WJ. Next-generation DNA sequencing techniques. New Biotechnol. 2009;25: 195–203. 10.1016/j.nbt.2008.12.009 19429539

[10] Sparks ME, Blackburn MB, Kuhar D, Gundersen-Rindal DE. Transcriptome of the *Lymantria dispar* (Gypsy Moth) larval midgut in response to infection by *Bacillus thuringiensis* . PLoS One. 2013;8: e61190 10.1371/journal.pone.0061190 23658687 PMC3641027

[11] Kumar A, Congiu L, Lindström L, Piiroinen S, Vidotto M, Grapputo A. Sequencing, *de Novo* assembly and annotation of the colorado potato beetle, *Leptinotarsa decemlineata*, transcriptome. PLoS One. 2014;9: e86012 10.1371/journal.pone.0086012 24465841 PMC3900453

[12] Poelchau MF, Reynolds JA, Denlinger DL, Elsik CG, Armbruster PA. A *de novo* transcriptome of the Asian tiger mosquito, *Aedes albopictus*, to identify candidate transcripts for diapause preparation. BMC Genomics. 2011;12: 619 10.1186/1471-2164-12-619 22185595 PMC3258294

[13] Zhou SS, Sun Z, Ma W, Chen W, Wang MQ. *De novo* analysis of the *Nilaparvata lugens* (Stål) antenna transcriptome and expression patterns of olfactory genes. Comp Biochem Phys D. 2014;9: 31–39. 10.1016/j.cbd.2013.12.002 24440828

[14] Vandesompele J, De Preter K, Pattyn F, Poppe B, Van Roy N, De Paepe A, et al Accurate normalization of real-time quantitative RT-PCR data by geometric averaging of multiple internal control genes. Genome Biol. 2002;3: research0034 12184808 10.1186/gb-2002-3-7-research0034 PMC126239

[15] Eisner E. A technique whose time has come? The Leading Edge. 1997;16: 171–172.

[16] Andersen CL, Jensen JL, Ørntoft TF. Normalization of real-time quantitative reverse transcription-PCR data: a model-based variance estimation approach to identify genes suited for normalization, applied to bladder and colon cancer data sets. Cancer Res. 2004;64: 5245–5250. 15289330 10.1158/0008-5472.CAN-04-0496

[17] Shen GM, Jiang HB, Wang XN, Wang JJ. Evaluation of endogenous references for gene expression profiling in different tissues of the oriental fruit fly *Bactrocera dorsalis* (Diptera: Tephritidae). BMC Mol Biol. 2010;11: 76 10.1186/1471-2199-11-76 20923571 PMC2972281

[18] Li R, Xie W, Wang S, Wu Q, Yang N, Yang X, et al Reference gene selection for qRT-PCR analysis in the sweetpotato whitefly, *Bemisia tabaci* (Hemiptera: Aleyrodidae). PLoS One. 2013;8: e53006 10.1371/journal.pone.0053006 23308130 PMC3540095

[19] Lu Y, Yuan M, Gao X, Kang T, Zhan S, Wan H, et al Identification and validation of reference genes for gene expression analysis using quantitative PCR in *Spodoptera litura* (Lepidoptera: Noctuidae). PLoS One. 2013;8: e68059 10.1371/journal.pone.0068059 23874494 PMC3706614

[20] Homma T, Watanabe K, Tsurumaru S, Kataoka H, Imai K, Kamba M, et al G protein-coupled receptor for diapause hormone, an inducer of *Bombyx* embryonic diapause. Biochem Bioph Res Co. 2006;344: 386–393. 10.1016/j.bbrc.2006.03.085 16600181

[21] Mitsumasu K, Ohta H, Tsuchihara K, Asaoka K, Ozoe Y, Niimi T, et al Molecular cloning and characterization of cDNAs encoding dopamine receptor-1 and -2 from brain-suboesophageal ganglion of the silkworm, *Bombyx mori* . Insect Mol Biol. 2008;17: 185–195. 10.1111/j.1365-2583.2008.00792.x 18353107

[22] Rubio RO, Suzuki A, Mitsumasu K, Homma T, Niimi T, Yamashita O, et al Cloning of cDNAs encoding sorbitol dehydrogenase-2a and b, enzymatic characterization, and up-regulated expression of the genes in *Bombyx mori* diapause eggs exposed to 5°C. Insect Biochem Molec. 2011;41: 378–387. 10.1016/j.ibmb.2011.02.007 21377527

[23] Fu W, Xie W, Zhang Z, Wang S, Wu Q, Liu Y, et al Exploring valid reference genes for quantitative real-time PCR analysis in *Plutella xylostella* (Lepidoptera: Plutellidae). Int J Biol Sci. 2013;9: 792–802. 10.7150/ijbs.5862 23983612 PMC3753443

[24] Teng X, Zhang Z, He G, Yang L, Li F. Validation of reference genes for quantitative expression analysis by real-time RT-PCR in four lepidopteran insects. J Insect Sci. 2012;12: 60 10.1673/031.012.6001 22938136 PMC3481461

[25] Radonić A, Thulke S, Mackay IM, Landt O, Siegert W, Nitsche A. Guideline to reference gene selection for quantitative real-time PCR. Biochem Bioph Res Co. 2004;313: 856–862. 10.1016/j.bbrc.2003.11.177 14706621

[26] Shi XQ, Guo WC, Wan PJ, Zhou LT, Ren XL, Ahmat T, et al Validation of reference genes for expression analysis by quantitative real-time PCR in *Leptinotarsa decemlineata* (Say). BMC Res Notes. 2013;6: 93 10.1186/1756-0500-6-93 23497596 PMC3600673

[27] Pfaffl MW, Tichopad A, Prgomet C, Neuvians TP. Determination of stable housekeeping genes, differentially regulated target genes and sample integrity: BestKeeper—Excel-based tool using pair-wise correlations. Biotechnol lett. 2004;26: 509–515. 15127793 10.1023/b:bile.0000019559.84305.47

[28] Xie F, Sun G, Stiller JW, Zhang B. Genome-wide functional analysis of the cotton transcriptome by creating an integrated EST database. PLoS One. 2011;6: e26980 10.1371/journal.pone.0026980 22087239 PMC3210780

[29] Ma CH, Ding N, Wang XP, Lei CL. Examination of parental effect on the progeny diapause by reciprocal cross test in the cabbage beetle, *Colaphellus bowringi* . J Insect Sci. 2011;11: 145 10.1673/031.011.14501 22224544 PMC3281385

[30] Grabherr MG, Haas BJ, Yassour M, Levin JZ, Thompson DA, Amit I, et al Full-length transcriptome assembly from RNA-Seq data without a reference genome. Nat Biotech. 2011;29: 644–652. 10.1038/nbt.1883 21572440 PMC3571712

[31] Kanehisa M, Goto S, Kawashima S, Okuno Y, Hattori M. The KEGG resource for deciphering the genome. Nucleic Acids Res. 2004;32: D277–D280. 14681412 10.1093/nar/gkh063 PMC308797

[32] Pfaffl MW. A new mathematical model for relative quantification in real-time RT–PCR. Nucleic Acids Res. 2001;29: e45 11328886 10.1093/nar/29.9.e45 PMC55695

[33] Poelchau MF, Reynolds JA, Elsik CG, Denlinger DL, Armbruster PA. Deep sequencing reveals complex mechanisms of diapause preparation in the invasive mosquito, *Aedes albopictus* . P Roy Soc B-Biol Sci. 2013;280: 20130143 10.1098/rspb.2013.0143 23516243 PMC3619507

[34] Xue J, Bao YY, Li B, Cheng YB, Peng ZY, Liu H, et al Transcriptome analysis of the brown planthopper *Nilaparvata lugens* . PLoS One. 2010;5: e14233 10.1371/journal.pone.0014233 21151909 PMC2997790

[35] Zhu JY, Li YH, Yang S, Li QW. *De novo* assembly and characterization of the global transcriptome for *Rhyacionia leptotubula* using Illumina paired-end sequencing. PLoS One. 2013;8: e81096 10.1371/journal.pone.0081096 24278383 PMC3837686

[36] Sim C, Denlinger DL. Insulin signaling and FOXO regulate the overwintering diapause of the mosquito *Culex pipiens* . Proc Natl Acad Sci U S A. 2008;105: 6777–6781. 10.1073/pnas.0802067105 18448677 PMC2373331

[37] Kim M, Denlinger DL. A potential role for ribosomal protein S2 in the gene network regulating reproductive diapause in the mosquito *Culex pipiens* . J Comp Physiol B. 2010;180: 171–178. 10.1007/s00360-009-0406-9 19774386

[38] de Boer ME, de Boer TE, Mariën J, Timmermans MJ, Nota B, van Straalen N, et al Reference genes for qRT-PCR tested under various stress conditions in *Folsomia candida* and *Orchesella cincta* (Insecta, Collembola). BMC Mol Biol. 2009;10: 54 10.1186/1471-2199-10-54 19486513 PMC2698932

[39] Van Hiel MB, Van Wielendaele P, Temmerman L, Van Soest S, Vuerinckx K, Huybrechts R, et al Identification and validation of housekeeping genes in brains of the desert locust *Schistocerca gregaria* under different developmental conditions. BMC Mol Biol. 2009;10: 56 10.1186/1471-2199-10-56 19508726 PMC2700112

[40] Ponton F, Chapuis MP, Pernice M, Sword GA, Simpson SJ. Evaluation of potential reference genes for reverse transcription-qPCR studies of physiological responses in *Drosophila melanogaster* . J Insect Physiol. 2011;57: 840–850. 10.1016/j.jinsphys.2011.03.014 21435341

[41] Paim RM, Pereira MH, Di Ponzio R, Rodrigues JO, Guarneri AA, Gontijo N, et al Validation of reference genes for expression analysis in the salivary gland and the intestine of *Rhodnius prolixus* (Hemiptera, Reduviidae) under different experimental conditions by quantitative real-time PCR. BMC Res Notes. 2012;5: 128 10.1186/1756-0500-5-128 22395020 PMC3337225

[42] Chapuis MP, Tohidi-Esfahani D, Dodgson T, Blondin L, Ponton F, Cullen D, et al Assessment and validation of a suite of reverse transcription-quantitative PCR reference genes for analyses of density-dependent behavioural plasticity in the Australian plague locust. BMC Mol Biol. 2011;12: 7 10.1186/1471-2199-12-7 21324174 PMC3048552

[43] Rajarapu SP, Mamidala P, Mittapalli O. Validation of reference genes for gene expression studies in the emerald ash borer (*Agrilus planipennis*). Insect Sci. 2012;19: 41–46.

[44] Scharlaken B, de Graaf DC, Goossens K, Brunain M, Peelman LJ, Jacobs FJ. Reference gene selection for insect expression studies using quantitative real-time PCR: The head of the honeybee, *Apis mellifera*, after a bacterial challenge. J Insect Sci. 2008;8: 33.

