# Supplementary figures and images for: A De Novo Transcriptome and Valid Reference Genes for Quantitative Real-Time PCR in Colaphellus bowringi
Source: PLoS One. 2015 Feb 18;10(2):e0118693. doi: 10.1371/journal.pone.0118693 (PMC4334893; doi:10.1371/journal.pone.0118693)

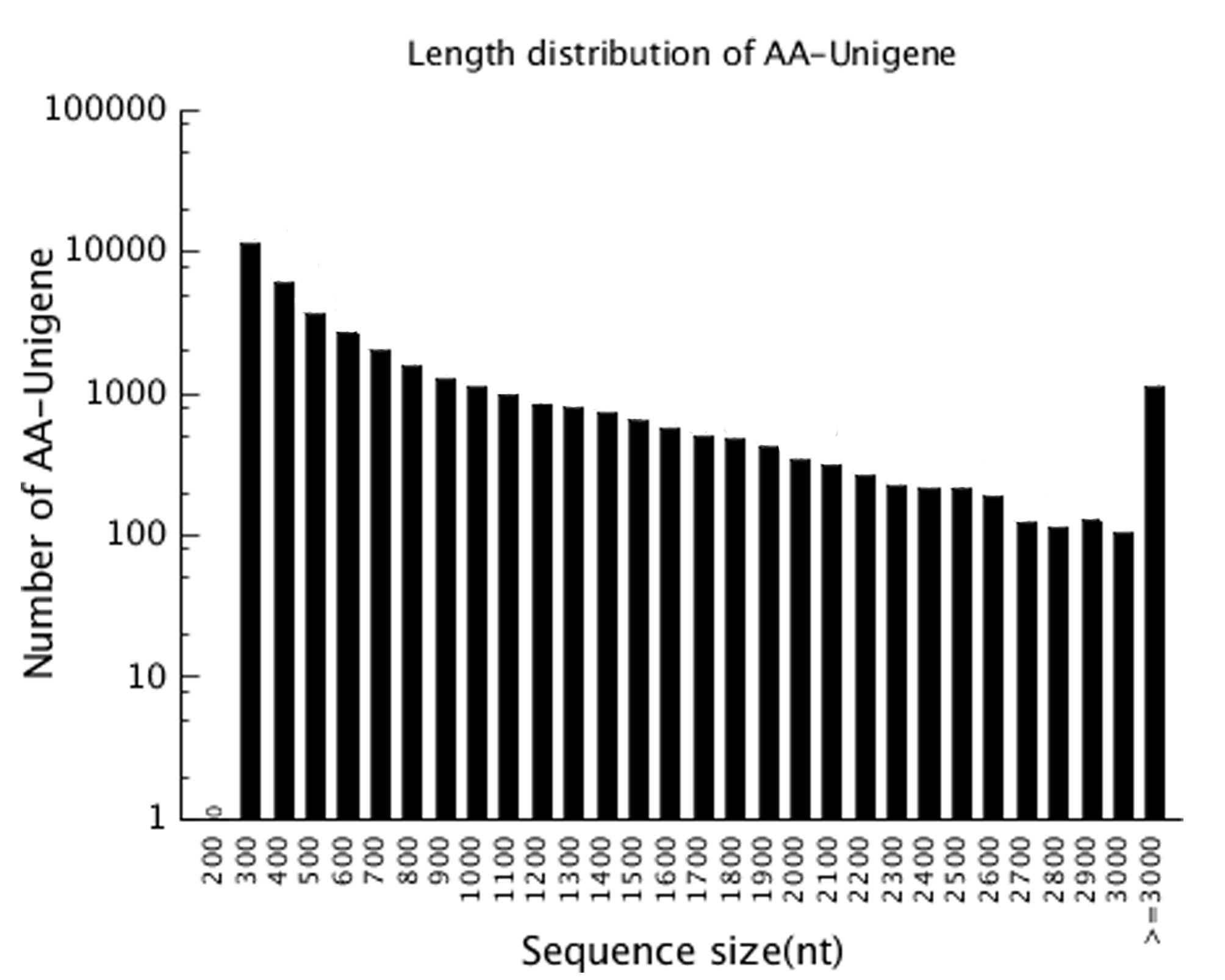

Supplement: S1 Fig — (TIF) [file pone.0118693.s001.tif]
