# Supplementary material for: A De Novo Transcriptome and Valid Reference Genes for Quantitative Real-Time PCR in Colaphellus bowringi
Source: PLoS One. 2015 Feb 18;10(2):e0118693. doi: 10.1371/journal.pone.0118693 (PMC4334893; doi:10.1371/journal.pone.0118693)
Supplement: S1 Table — (DOC) [file pone.0118693.s002.doc]

**S1 Table. Primers used for RT-PCR analysis to identify the accuracy of the reference genes of *Colaphellus bowringi*.**

| **Gene name (Abbreviation)** | **Gene number** | **Accession number** | **Primer sequence (5'-3')^1^** |
| --- | --- | --- | --- |
| **Glyceraldehyde-3-phosphate dehydrogenase**  (*GAPDH*) | Unigene1519 | KJ534558 | F: ATCACGTGACCAGCCGATTT |
|  |  |  | R:GTGACTAAGCCACAGCTCAGA |
| **Ribosomal protein L32e**  (*RPL32e*) | Unigene1573 | KJ534560 | F: AAGACAACAGTTGGCAACGC |
|  |  |  | R:TTGATGAAAACACATTCTGAGGTT |
| **Ribosomal protein L19**  (*RPL19*) | Unigene1605 | KJ534559 | F: GGCGCTACTGTCTAGTGAAGT |
|  |  |  | R: AACATCTTGGCACGAGCCTT |
| **Elongation factor-1 α**  (*EF1α*) | CL1951.Contig1 | KJ534557 | F: GACCGCGGATTATCGTCCTT |
|  |  |  | R: GTCTGCACTCCAGCTCTGTT |
| **TATA-Box binding protein**  (*TBP*) | Unigene6272 | KJ534562 | F: ACCCAGTATTGGGACACCTCT |
|  |  |  | R: ACTTTAGCACCCGTCAACACT |
| **TATA-Box binding protein 1**  (*TBP1*) | Unigene8370 | KJ534561 | F: TGAGGCTCGGATGAAGTTGT |
|  |  |  | R: CACGATGCCATAACCTTCGC |
| **Actin1**  (*ACT1*) | Unigene2726 | KJ534552 | F: ACTGAATCGCCGTAGGTGTG |
|  |  |  | R: TGTCATCGTTCCAAACACTGGA |
| **Actin2**  (*ACT2*) | CL328.Contig1 | KJ534553 | F: TGAGGTGTGGTGTATTGTTGACT |
|  |  |  | R: ATTTGCCCACCCTGTACCAC |
| **α-tubulin**  (*αTUB*) | Unigene2458 | KJ534555 | F: GCTCTACTGCTTGGAGCACA |
|  |  |  | R: TAATGCTGCCAGGTCTTCCC |
| **α-tubulin1**  (*αTUB1*) | CL1629.Contig2 | KJ534554 | F: TTAGGCGTTGGTTGGTGACG |
|  |  |  | R: TGGTTCGAGTGAGCCCTTTC |
| **β-tubulinC**  (*βTUBC*) | Unigene3265 | KJ534556 | F: CCGAAAGTCATCTTCAACTCCCT |
|  |  |  | R: ATCTTCACGACAATTATCCCACC |

“1”: F, forward primer; R, reverse primer.
