# Supplementary material for: A De Novo Transcriptome and Valid Reference Genes for Quantitative Real-Time PCR in Colaphellus bowringi
Source: PLoS One. 2015 Feb 18;10(2):e0118693. doi: 10.1371/journal.pone.0118693 (PMC4334893; doi:10.1371/journal.pone.0118693)
Supplement: S3 Table — (DOC) [file pone.0118693.s004.doc]

**S3 Table. Primers used for qRT-PCR analysis to exploring valid reference genes of *Colaphellus bowringi*.**

| **Gene name (Abbreviation)** | **Primer Sequence (5'-3')^1^** | **Amplicon size (bp)** | **Tm(℃)^2^** | **E(%)^3^** | **(R^2^)^4^** |
| --- | --- | --- | --- | --- | --- |
| **Glyceraldehyde-3-phosphate dehydrogenase**  (*GAPDH*) | F:ACGTGGTGCATCACAGAACA | 100 | 60 | 105.4 | 0.998 |
|  | R:GGCCATTCCGGTAAGTTTGC |  |  |  |  |
| **Ribosomal protein L32e**  (*RPL32e*) | F:GCTCATGCAGAACCGCAAAT | 126 | 60 | 100 | 0.998 |
|  | R:CGTAGCCTAGCGTGTCCATT |  |  |  |  |
| **Ribosomal protein L19**  (*RPL19*) | F:GTAATGCGATGCGGCAAGAA | 137 | 60 | 102.2 | 0.998 |
|  | R:GAGTGCACCGCTACAGGTTT |  |  |  |  |
| **Elongation factor-1 α**  (*EF1α*) | F:CTTGGTACCATCCAAGCCCA | 108 | 60 | 106.4 | 0.998 |
|  | R:TTGATGACACCTACAGCGACC |  |  |  |  |
| **TATA-Box binding protein**  (*TBP*) | F:GAGAATTCGGGAACCTCGCA | 94 | 60 | 102.5 | 0.996 |
|  | R:TGCTAGCCGAGAGTCTTCCT |  |  |  |  |
| **TATA-Box binding protein 1**  (*TBP1*) | F:TGAACCAGAACTCCACCCAG | 115 | 60 | 108.2 | 0.996 |
|  | R:TTGGACATCAGCTACGCTCG |  |  |  |  |
| **Actin1**  (*ACT1*) | F:TCAAGCGGTGTTAGCTCTGG | 80 | 60 | 100.2 | 0.996 |
|  | R:TATCGATCACGATGCCGGTG |  |  |  |  |
| **Actin2**  (*ACT2*) | F:ATCCGCATCGAAGATCCACC | 126 | 60 | 102.7 | 0.998 |
|  | R:CACGCCACCCTCCTCATATT |  |  |  |  |
| **α-tubulin**  (*αTUB*) | F:TGCCCAAGGATGTGAATGCT | 126 | 60 | 102.4 | 0.998 |
|  | R:CATTTGGCACAACAGTCGGG |  |  |  |  |
| **α-tubulin1**  (*αTUB1*) | F:CGCCAACAACTATGCTCGTG | 113 | 60 | 102.2 | 0.997 |
|  | R:TGAGGAAACCTTGCAGTCCG |  |  |  |  |
| **β-tubulinC**  (*βTUBC*) | F:TCCGCAAAGATGGAAGTGGA | 104 | 60 | 102.3 | 0.997 |
|  | R:AACTGCAGAGTGTTTCTGCG |  |  |  |  |

“1”: F, forward primer; R, reverse primer; “2”: Tm, Annealing temperature; “3”: E, Efficiency; “4”: R2, Coefficient of determination.
