# Supplementary material for: A De Novo Transcriptome and Valid Reference Genes for Quantitative Real-Time PCR in Colaphellus bowringi
Source: PLoS One. 2015 Feb 18;10(2):e0118693. doi: 10.1371/journal.pone.0118693 (PMC4334893; doi:10.1371/journal.pone.0118693)
Supplement: S4 Table — (DOC) [file pone.0118693.s005.doc]

**S4 Table**. **Ranking of candidate reference genes in response to combination samples evaluated by Normfinder of *Colaphellus bowringi*.**

| **Rank** | **Pho. VS Tissue^1^** | | | |  | **Pho. VS Strain^2^** | | | |  | **Pho. VS 24 h Pho. of 4-day-old Larva ^3^** | | | |
| --- | --- | --- | --- | --- | --- | --- | --- | --- | --- | --- | --- | --- | --- | --- |
|  | **Gene** | **SV1^4^** | **Best two genes^5^** | **SV2^6^** |  | **Gene** | **SV1** | **Best two genes** | **SV2** |  | **Gene** | **SV1** | **Best two genes** | **SV2** |
| 1 | *1ACT* | 0.211 | ***TBP1*** | 0.112 |  | *βTUBC* | 0.006 | ***EF1α*** | 0.006 |  | *RPL19* | 0.01 | ***RPL19*** | 0.013 |
| 2 | *αTUB1* | 0.341 | ***αTUB1*** |  |  | *EF1α* | 0.009 | ***βTUBC*** |  |  | *αTUB1* | 0.023 | ***αTUB1*** |  |
| 3 | *βTUBC* | 0.355 |  |  |  | *TBP* | 0.053 |  |  |  | *βTUBC* | 0.026 |  |  |
| 4 | *TBP1* | 0.396 |  |  |  | *ACT1* | 0.073 |  |  |  | *RPL32e* | 0.039 |  |  |
| 5 | *2ACT* | 0.433 |  |  |  | *RPL19* | 0.074 |  |  |  | *EF1α* | 0.043 |  |  |
| 6 | *EF1α* | 0.442 |  |  |  | *TBP1* | 0.08 |  |  |  | *TBP* | 0.048 |  |  |
| 7 | *RPL19* | 0.507 |  |  |  | *αTUB1* | 0.081 |  |  |  | *TBP1* | 0.054 |  |  |
| 8 | *RPL32e* | 0.507 |  |  |  | *RPL32e* | 0.12 |  |  |  | *ACT1* | 0.093 |  |  |
| 9 | *TBP* | 0.515 |  |  |  | *GAPDH* | 0.13 |  |  |  | *GAPDH* | 0.146 |  |  |
| 10 | *GAPDH* | 0.653 |  |  |  | *ACT2* | 0.151 |  |  |  | *ACT2* | 0.18 |  |  |
| 11 | *αTUB* | 1.717 |  |  |  | *αTUB* | 0.399 |  |  |  | *αTUB* | 0.266 |  |  |

“1”: Pho. VS Tissue means photoperiod combination with tissue; “2”: Pho. VS Strain means photoperiod combination with strain; “3”: Pho. VS 24 h Pho. of 4-day-old Larva means photoperiod combination with 24 h photoperiod of 4-day-old larva; “4”: Stability Value was evaluated by Normfinder. “5”: Best combination of two genes; “6”: Stability value for best combination of two genes evaluated by Normfinder.
