# Supplementary material for: A De Novo Transcriptome and Valid Reference Genes for Quantitative Real-Time PCR in Colaphellus bowringi
Source: PLoS One. 2015 Feb 18;10(2):e0118693. doi: 10.1371/journal.pone.0118693 (PMC4334893; doi:10.1371/journal.pone.0118693)
Supplement: S5 Table — (DOC) [file pone.0118693.s006.doc]

**S5 Table. The statistical results of the candidate reference genes of *Colaphellus bowringi* calculated by BestKeeper.**

| **Genes** | ***GAPDH*** | ***RPL32e*** | ***RPL19*** | ***EF1α*** | ***TBP*** | ***TBP1*** | ***ACT1*** | ***ACT2*** | ***αTUB*** | ***αTUB1*** | ***βTUBC*** |
| --- | --- | --- | --- | --- | --- | --- | --- | --- | --- | --- | --- |
| ***Development Stage*** | | | | | | | | | | | |
| n | 8 | 8 | 8 | 8 | 8 | 8 | 8 | 8 | 8 | 8 | 8 |
| GM(Ct)^1^ | 17.84 | 16.68 | 17.19 | 16.21 | 23.69 | 23.31 | 23.57 | 23.77 | 23.74 | 18.43 | 22.2 |
| SD(Ct)^2^ | 0.452 | 0.522 | 0.483 | 0.43 | 0.502 | 0.448 | 0.285 | 0.414 | 4.583 | 0.447 | 0.396 |
| Rank | 7 | 10 | 8 | 4 | 9 | 6 | 1 | 3 | 11 | 5 | 2 |
| ***Tissue*** | | | | | | | | | | | |
| n | 4 | 4 | 4 | 4 | 4 | 4 | 4 | 4 | 4 | 4 | 4 |
| GM(Ct) | 18.14 | 17.32 | 17.79 | 16.48 | 24.71 | 24.14 | 24.2 | 23.63 | 23.99 | 19.6 | 22.95 |
| SD(Ct) | 0.343 | 0.376 | 0.366 | 0.361 | 1.214 | 1.09 | 0.465 | 0.384 | 3.651 | 0.37 | 0.406 |
| Rank | 1 | 5 | 3 | 2 | 10 | 9 | 8 | 6 | 11 | 4 | 7 |
| ***Sex*** | | | | | | | | | | | |
| n | 2 | 2 | 2 | 2 | 2 | 2 | 2 | 2 | 2 | 2 | 2 |
| GM(Ct) | 17.8 | 17.21 | 17.74 | 16.71 | 24.17 | 23.74 | 23.7 | 23.67 | 24.42 | 18.7 | 22 |
| SD(Ct) | 0.356 | 0.676 | 0.641 | 0.628 | 0.52 | 0.469 | 0.031 | 0.019 | 4.078 | 0.17 | 0.278 |
| Rank | 5 | 10 | 9 | 8 | 7 | 6 | 2 | 1 | 11 | 3 | 4 |
| ***Strain*** | | | | | | | | | | | |
| n | 2 | 2 | 2 | 2 | 2 | 2 | 2 | 2 | 2 | 2 | 2 |
| GM(Ct) | 17.34 | 16.25 | 16.88 | 16.1 | 23.69 | 23.38 | 23.65 | 23.68 | 21.06 | 18.62 | 21.71 |
| SD(Ct) | 0.103 | 0.296 | 0.228 | 0.006 | 0.035 | 0.102 | 0.016 | 0.013 | 0.382 | 0.244 | 0.017 |
| Rank | 7 | 10 | 8 | 1 | 5 | 6 | 3 | 2 | 11 | 9 | 4 |
| ***Photoperiod*** | | | | | | | | | | | |
| n | 2 | 2 | 2 | 2 | 2 | 2 | 2 | 2 | 2 | 2 | 2 |
| GM(Ct) | 17.41 | 16.92 | 17.4 | 16.33 | 24.05 | 23.67 | 23.73 | 23.56 | 21.57 | 19.18 | 21.93 |
| SD(Ct) | 0.038 | 0.376 | 0.29 | 0.238 | 0.402 | 0.392 | 0.067 | 0.126 | 0.907 | 0.312 | 0.203 |
| Rank | 1 | 8 | 6 | 5 | 10 | 9 | 2 | 3 | 11 | 7 | 4 |
| ***24 h Photoperiod of 4-day-old Larva*** | | | | | | | | | | | |
| n | 7 | 7 | 7 | 7 | 7 | 7 | 7 | 7 | 7 | 7 | 7 |
| GM(Ct) | 17.82 | 17.13 | 17.54 | 16.75 | 24.71 | 23.86 | 23.77 | 23.39 | 28.89 | 17.81 | 22.2 |
| SD(Ct) | 0.219 | 0.117 | 0.083 | 0.147 | 0.074 | 0.172 | 0.078 | 0.127 | 0.202 | 0.118 | 0.171 |
| Rank | 11 | 4 | 3 | 7 | 1 | 9 | 2 | 6 | 10 | 5 | 8 |

“1”: GM(Ct), geometric mean of Ct value; “2”: SD(Ct), standard deviation of Ct value
